# Synthesis and crystal structure of hydrated μ-oxa­lato-bis­{bis­[3-methyl-5-(pyridin-2-yl)-1*H*-1,2,4-triazole]iron(II)} bis­(toluene­sulfonate) 2.75-hydrate

**DOI:** 10.1107/S2056989022007460

**Published:** 2022-07-26

**Authors:** Yuliia P. Petrenko, Yurii S. Bibik, Dmytro M. Khomenko, Roman O. Doroshchuk, Il‘ya A. Gural’skiy, Sergiu Shova, Rostyslav D. Lampeka, Ilona V. Raspertova

**Affiliations:** a Department of Chemistry, Kyiv National Taras Shevchenko University, Volodymyrska, st. 64, Kyiv, Ukraine; b Enamine Ltd., Chervonotkatska Street 78, Kyiv 02094, Ukraine; c "Petru Poni" Institute of Macromolecular Chemistry, Aleea Gr. Ghica, Voda 41A, 700487 Iasi, Romania; Katholieke Universiteit Leuven, Belgium

**Keywords:** crystal structure, iron(II) complex, 1,2,4-triazole, oxalato-bridged complex, X-ray crystallography

## Abstract

In the title compound [Fe_2_(C_2_O_4_)(C_8_H_8_N_4_)_4_](CH_3_C_6_H_4_SO_3_)_2_·2.75H_2_O, the two Fe^II^ ions have a highly distorted octa­hedral FeN_4_O_2_ environment formed by two bidentate triazole-based chelating ligands and a bis-bidentate oxalate bridging anion that connects the metal ions. Stabilization within the crystal structure is provided *via* a system of O—H⋯O and N—H⋯O hydrogen bonding, which determines the formation of a two-dimensional architecture along the *a-*axis direction.

## Abstract 2

A bis-bidentate oxalate bridging anion connects two Fe^II^ ions further surrounded by bidentate pyridyl-triazole ligands.

## 1. Chemical context

The study of coordination compounds based on substituted 1,2,4-triazoles and 3*d* and 4*d* transition metals allows the design of supra­molecular structures that can find applications in various fields such as mol­ecular magnetism, catalysis, electrochemistry or cluster engineering (Zhang *et al.*, 2017 ▸; Zakharchenko *et al.*, 2019 ▸; Chen *et al.*, 2015 ▸; Petrenko *et al.*, 2020 ▸, 2021 ▸). The presence of the pyridine ring in such triazole systems leads to the formation of inter­esting isolated metal–organic frameworks that demonstrate promising magnetic properties, making them suitable for application as mol­ecule-based magnets (Yao *et al.*, 2015 ▸; Han *et al.*, 2017 ▸; Li *et al.*, 2015 ▸; Huang *et al.*, 2015 ▸). Moreover, a combination of 3*d* ^4^–3*d* ^7^ metals with N-donor bridging ligands may form coordination compounds with switchable spin states (Aromí *et al.*, 2011 ▸; Kucheriv *et al.*, 2021 ▸). This phenomenon is called spin crossover. Changes in the external temperature, pressure, magnetic field, light radiation or the presence of a guest alters the magnetic, electrical, mechanical and optical properties significantly in these compounds (Gütlich & Goodwin, 2004 ▸). Therefore, the synthesis and crystallographic characterization of these complexes are of current inter­est.

On the other hand, the ability of the oxalate anion to generate homobinuclear complexes is well known (Craig *et al.*, 2010 ▸; Selmi *et al.*, 2021 ▸; Karimpour *et al.*, 2013 ▸; Paine *et al.*, 2007 ▸). The coordination chemistry of oxalato-bridged binuclear Fe^II^ complexes with pyridyl-triazole chelating ligands is less studied. A few examples with a similar type of ligand indicate that complexes of this kind possess inter­esting magnetic and oxidizing properties (de Ruiter *et al.*, 2008 ▸; Oliveira *et al.*, 2018 ▸). In order to continue research in this field and in the course of our studies dedicated to the investigation of triazoles and, in particular, 3-methyl-5-(pyrid-2-yl)-2*H*-1,2,4-triazole (metrzpy) (Zakharchenko *et al.*, 2017 ▸; Zakharchenko, Khomenko, Doroschuk, Raspertova, Fesych *et al.*, 2021 ▸; Zakharchenko, Khomenko, Doroshchuk, Raspertova, Shova *et al.*, 2021 ▸), we report herein the synthesis and crystal structure of a new binuclear iron(II) complex with this ligand. 
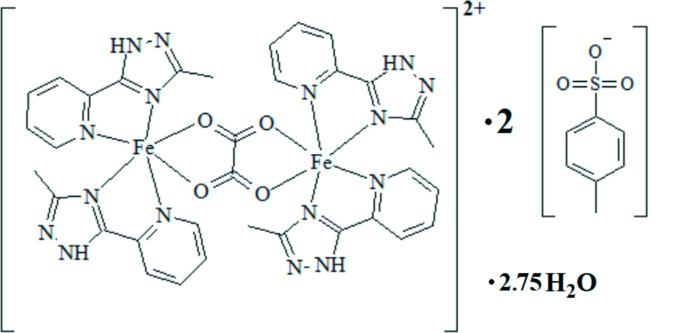


## 2. Structural commentary

**Figure 1:**
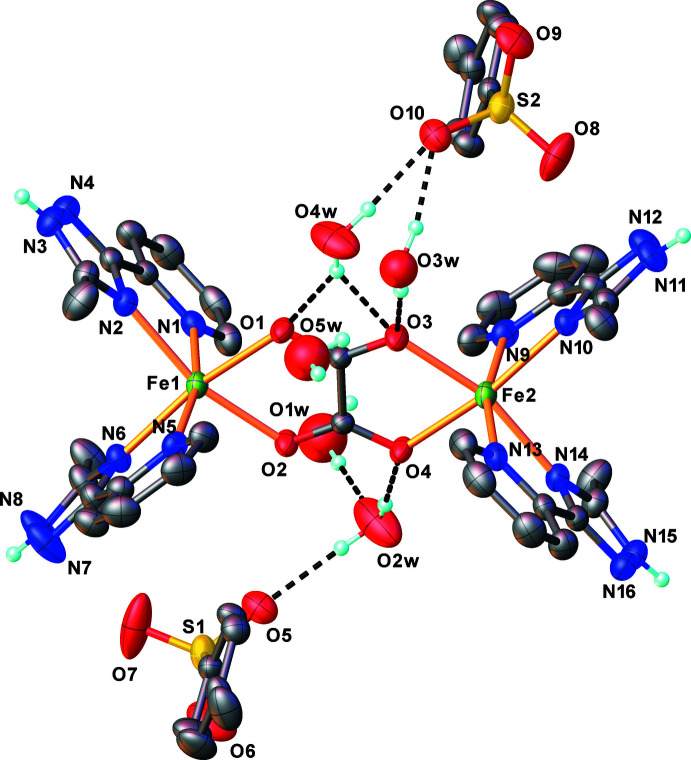
X-ray mol­ecular structure of the title compound with selected atom labels and displacement ellipsoids drawn at the 50% level. Some H atoms are omitted for clarity. Key: carbon, grey; nitrogen, blue; oxygen, red; sulfur, yellow; iron, light green.

The structure of the title compound is built up from dinuclear [Fe_2_(metrzpy)_4_(C_2_O_4_)]^2+^ complex cations, *p*-toluene­sulfonate anions and co-crystallized water mol­ecules in a 1:2:2.75 ratio. It crystallizes in the triclinic space group *P* 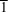 with two complex mol­ecules per unit cell. Each iron(II) ion has an N_4_O_2_ coordination environment in a distorted octa­hedral geometry provided by two chelating metrzpy ligands in *cis* positions and a bidentate bridging oxalate anion (Fig. 1 ▸, Table 1 ▸). The reduced values of the angles subtended at the iron atom by the metrzpy and oxalate ligands are the main factors behind this distortion. The Fe—N and Fe—O bond lengths vary in the ranges 2.150 (3)–2.209 (3) Å and 2.123 (2)–2.171 (2) Å, respectively. The Fe1⋯Fe2 separation across the oxalate bridge of 5.576 (6) Å is in good agreement with previously reported values for other oxalate-bridged iron(II) complexes. The sets of coordinating atoms (O1/O2/N2/N6 for Fe1 and O3/O4/N10/N14 for Fe2) defining the mean equatorial planes are co-planar within 0.22 and 0.20 Å, while the displacement of the metal atom from these planes is 0.015 (1) and 0.037 (1) Å, respectively. The dihedral angle formed by each plane and the mean plane of the oxalate atoms is of 9.74 (6)° for Fe1 and 10.04 (7)° for Fe2.

## 3. Supra­molecular features

**Figure 2:**
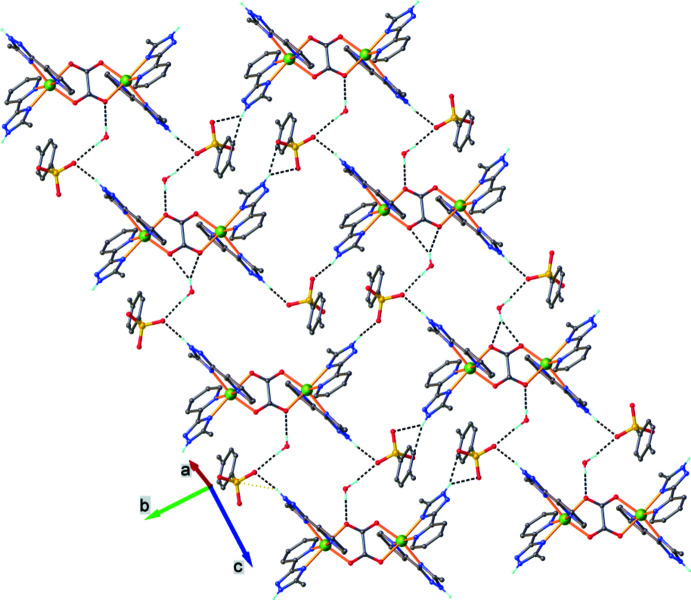
Two-dimensional supra­molecular network viewed along the *a* axis.

**Figure 3:**
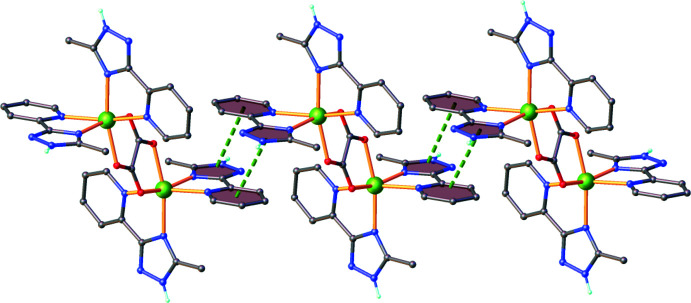
*π-*-*π* stacking between adjacent complex cations. Centroid-to-centroid contacts are shown as green dashed lines.

All the species present in the structure are inter­connected *via* a system of O—H⋯O and N—H⋯O hydrogen bonds (Table 2 ▸), which determines the formation of a two-dimensional architecture, as shown in Fig. 2 ▸. Further analysis has shown that the main crystal-structure motif consists of the parallel packing of 2D layers consolidated by the *π*–*π* stacking inter­actions observed between triazole and pyridine rings of adjacent cationic entities (Fig. 3 ▸) with a centroid-to-centroid distance of 3.746 (1) Å.

## 4. Database survey

A search of the Cambridge Structural Database (CSD, version 5.43, last update November 2021; Groom *et al.*, 2016 ▸) gave 189 hits for the Fe_2_(μ-C_2_O_4_) unit, the majority of which are iron(II)-based metal–organic coordination polymers. Besides them, there are several homobimetallic structures with an [FeN_4_O_2_] coordination environment: AVIMUN (Spek *et al.*, 2004 ▸), LOZHOA (Oliveira *et al.*, 2018 ▸), NOLSUF and NOLTAM (Gusev *et al.*, 2019 ▸) and VIHCIZ (Paine *et al.*, 2007 ▸). It must be noted that AVIMUN is a homologue of the title compound and contains a 3-ethyl-1,2,4-triazole fragment; however, it has a different packing and the crystal structure belongs to the monoclinic system.

A search for the structures of coordination compounds based on 3-methyl-5-(pyrid-2-yl)-2*H*-1,2,4-triazole revealed ten hits. Three of these structures represent our previous studies: CAMSUI (Zakharchenko, Khomenko, Doroschuk, Raspertova, Shova *et al.*, 2021 ▸), IXIBID and IXIBOJ (Petrenko *et al.*, 2021 ▸). The other structures correspond to mixed-ligand complexes with various metals, among them: NIYRAQ (Cao *et al.*, 2014 ▸), QURBIQ (Guetlich & Schollmeyer, 2015 ▸), REWSOC (Cheng *et al.*, 2007 ▸), SARQIO (Muller *et al.*, 2013 ▸) and VESZOI (Buchanan *et al.*, 1990 ▸).

## 5. Synthesis and crystallization

The triazole ligand was prepared according to a synthesis described in the literature (Zakharchenko *et al.*, 2017 ▸). Single crystals of [Fe_2_(C_2_O_4_)(metrzpy)_4_](CH_3_C_6_H_4_SO_3_)_2_·2.75H_2_O were obtained by the liquid-to-liquid diffusion technique using a layering tube. The bottom was filled with Fe(CH_3_C_6_H_4_SO_3_)_2_·6H_2_O (50.6 mg, 0.1 mmol) in 2 ml of water. The middle was filled with a solution of 2 ml methanol/water (1:1) containing ascorbic acid (35.2 mg, 0.2 mmol). Then the top was filled with a solution of metrzpy ligand (32.0 mg, 0.2 mmol) in 2 ml of methanol. Afterwards, the tube was sealed with parafilm and light brown square-plate single crystals were formed within 3 days in relative high yield (*ca* 50%).

## 6. Refinement

Crystal data, data collection and structure refinement details are summarized in Table 3 ▸. All hydrogen atoms were placed geometrically and refined as riding, with C—H = 0.96 (CH_3_), 0.93 Å (C_arom_), N—H = 0.86 Å and O—H = 0.85–0.87Å, and with *U*_iso_(H) = 1.2*U*_eq_(C_arom_) or 1.5*U*_eq_(C-meth­yl). N-bound H atoms were refined with *U*_iso_(H) = 1.2*U*_eq_(N). The idealized OH_2_ mol­ecule was fixed using an AFIX 3, *U*_iso_(H) = 1.5*U*_eq_(O_water_).

## Supplementary Material

Crystal structure: contains datablock(s) I. DOI: 10.1107/S2056989022007460/vm2269sup1.cif

Structure factors: contains datablock(s) I. DOI: 10.1107/S2056989022007460/vm2269Isup2.hkl

CCDC reference: 2191587

Additional supporting information:  crystallographic information; 3D view; checkCIF report

## Figures and Tables

**Table 1:** Selected bond lengths (Å)

Fe1—O1	2.171 (2)	Fe2—O3	2.123 (2)
Fe1—O2	2.123 (2)	Fe2—O4	2.157 (2)
Fe1—N1	2.203 (3)	Fe2—N9	2.209 (3)
Fe1—N2	2.150 (3)	Fe2—N10	2.165 (3)
Fe1—N5	2.197 (3)	Fe2—N13	2.206 (3)
Fe1—N6	2.162 (3)	Fe2—N14	2.159 (3)

**Table 2:** Hydrogen-bond geometry (Å, °)

*D*—H⋯*A*	*D*—H	H⋯*A*	*D*⋯*A*	*D*—H⋯*A*
N3—H3⋯O10^i^	0.86	1.95	2.766 (4)	159
N7—H7⋯O6^ii^	0.86	2.34	3.064 (5)	142
N7—H7⋯O7^ii^	0.86	2.34	3.141 (6)	154
N11—H11⋯O9^iii^	0.86	1.92	2.769 (4)	170
N15—H15⋯O5^iv^	0.86	1.99	2.825 (4)	163
C4—H4⋯O2*W* ^v^	0.93	2.48	3.383 (5)	165
C11—H11*A*⋯O5*W*	0.93	2.49	3.206 (8)	134
C28—H28⋯O4*W* ^vi^	0.93	2.54	3.421 (6)	159
O2*W*—H2*WA*⋯O4	0.85	2.10	2.949 (4)	174
O2*W*—H2*WB*⋯O5	0.86	1.99	2.838 (4)	172
O4*W*—H4*WA*⋯O1	0.87	2.34	3.123 (5)	150
O4*W*—H4*WA*⋯O3	0.87	2.25	3.037 (4)	151
O4*W*—H4*WB*⋯O10	0.87	1.92	2.788 (5)	174
O5*W*—H5*WA*⋯O4*W* ^vi^	0.86	1.98	2.810 (11)	159
O5*W*—H5*WB*⋯O4*W*	0.86	2.28	2.850 (10)	123
C13—H13⋯O8^vi^	0.93	2.57	3.256 (5)	131
C21—H21⋯O7^v^	0.93	2.44	3.280 (6)	150

Symmetry codes: (i) 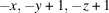 ; (ii) 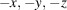 ; (iii) 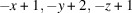 ; (iv) 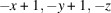 ; (v) 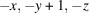 ; (vi) 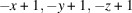 .

**Table 3:** Experimental details

Crystal data	
Chemical formula	[Fe_2_(C_2_O_4_)(C_8_H_8_N_4_)_4_](C_7_H_7_O_3_S)_2_·2.75H_2_O
*M*_r_	1232.37
Crystal system, space group	Triclinic, *P* 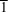
Temperature (K)	293
*a*, *b*, *c* (Å)	9.9635 (4), 14.4905 (6), 20.1131 (8)
α, β, γ (°)	96.736 (4), 101.490 (4), 95.216 (4)
*V* (Å^3^)	2806.5 (2)
*Z*	2
Radiation type	Mo *K*α
μ (mm^−1^)	0.67
Crystal size (mm)	0.35 × 0.2 × 0.15

Data collection	
Diffractometer	Rigaku Oxford Diffraction Xcalibur, Eos
Absorption correction	Multi-scan (*CrysAlis PRO*; Rigaku OD, 2021 ▸)
*T*_min_, *T*_max_	0.923, 1.000
No. of measured, independent and observed [*I* > 2σ(*I*)] reflections	20140, 9886, 7117
*R*_int_	0.031
(sin θ/λ)_max_ (Å^−1^)	0.595

Refinement	
*R*[*F* ^2^ > 2σ(*F* ^2^)], *wR*(*F* ^2^), *S*	0.058, 0.132, 1.06
No. of reflections	9886
No. of parameters	739
H-atom treatment	H-atom parameters constrained
Δρ_max_, Δρ_min_ (e Å^−3^)	0.59, −0.52

Computer programs: *CrysAlis PRO* (Rigaku OD, 2021 ▸), *SHELXT* (Sheldrick, 2015*a* ▸), *SHELXL2018/3* (Sheldrick, 2015*b* ▸) and *OLEX2* (Dolomanov *et al.*, 2009 ▸).

## supplementary crystallographic information

### Crystal data

**Table:**

[Fe_2_(C_2_O_4_)(C_8_H_8_N_4_)_4_](C_7_H_7_O_3_S)_2_·2.75H_2_O	*Z* = 2
*M**_r_* = 1232.37	*F*(000) = 1275
Triclinic, *P*1	*D*_x_ = 1.458 Mg m^−^^3^
*a* = 9.9635 (4) Å	Mo *K*α radiation, λ = 0.71073 Å
*b* = 14.4905 (6) Å	Cell parameters from 5996 reflections
*c* = 20.1131 (8) Å	θ = 2.0–26.2°
α = 96.736 (4)°	µ = 0.67 mm^−^^1^
β = 101.490 (4)°	*T* = 293 K
γ = 95.216 (4)°	Block, clear light brown
*V* = 2806.5 (2) Å^3^	0.35 × 0.2 × 0.15 mm

### Data collection

**Table:**

Rigaku Oxford Diffraction Xcalibur, Eos diffractometer	9886 independent reflections
Radiation source: fine-focus sealed X-ray tube, Enhance (Mo) X-ray Source	7117 reflections with *I* > 2σ(*I*)
Graphite monochromator	*R*_int_ = 0.031
Detector resolution: 8.0797 pixels mm^-1^	θ_max_ = 25.0°, θ_min_ = 1.7°
ω scans	*h* = −11→11
Absorption correction: multi-scan (CrysAlisPro; Rigaku OD, 2021)	*k* = −17→15
*T*_min_ = 0.923, *T*_max_ = 1.000	*l* = −23→23
20140 measured reflections	

### Refinement

**Table:**

Refinement on *F*^2^	Primary atom site location: dual
Least-squares matrix: full	Hydrogen site location: mixed
*R*[*F*^2^ > 2σ(*F*^2^)] = 0.058	H-atom parameters constrained
*wR*(*F*^2^) = 0.132	*w* = 1/[σ^2^(*F*_o_^2^) + (0.0447*P*)^2^ + 1.2466*P*] where *P* = (*F*_o_^2^ + 2*F*_c_^2^)/3
*S* = 1.06	(Δ/σ)_max_ < 0.001
9886 reflections	Δρ_max_ = 0.59 e Å^−^^3^
739 parameters	Δρ_min_ = −0.52 e Å^−^^3^
0 restraints	

### Special details

**Table:**

Geometry. All esds (except the esd in the dihedral angle between two l.s. planes) are estimated using the full covariance matrix. The cell esds are taken into account individually in the estimation of esds in distances, angles and torsion angles; correlations between esds in cell parameters are only used when they are defined by crystal symmetry. An approximate (isotropic) treatment of cell esds is used for estimating esds involving l.s. planes.

### Fractional atomic coordinates and isotropic or equivalent isotropic displacement parameters (Å^2^)

**Table:**

	*x*	*y*	*z*	*U*_iso_*/*U*_eq_	Occ. (<1)
Fe1	0.06627 (5)	0.33384 (3)	0.24663 (2)	0.04095 (15)	
Fe2	0.46059 (5)	0.63703 (3)	0.25705 (2)	0.04295 (15)	
O1	0.1636 (2)	0.46283 (16)	0.31108 (11)	0.0477 (6)	
O2	0.2035 (3)	0.38999 (16)	0.18929 (11)	0.0485 (6)	
O3	0.3255 (3)	0.58058 (16)	0.31534 (11)	0.0505 (6)	
O4	0.3654 (3)	0.50805 (16)	0.19378 (11)	0.0487 (6)	
N1	−0.1150 (3)	0.38744 (19)	0.18860 (13)	0.0445 (7)	
N2	−0.0855 (3)	0.3172 (2)	0.30813 (13)	0.0451 (7)	
N3	−0.2364 (3)	0.2948 (2)	0.36995 (14)	0.0553 (8)	
H3	−0.271736	0.279820	0.403417	0.066*	
N4	−0.3071 (3)	0.3274 (2)	0.31482 (15)	0.0540 (8)	
N5	0.2127 (3)	0.2521 (2)	0.30396 (13)	0.0462 (7)	
N6	0.0155 (3)	0.19564 (19)	0.18814 (13)	0.0440 (7)	
N7	−0.0330 (4)	0.0583 (2)	0.13003 (17)	0.0922 (14)	
H7	−0.071277	0.014005	0.097691	0.111*	
N8	0.0632 (4)	0.0470 (2)	0.18557 (17)	0.0848 (13)	
N9	0.3081 (3)	0.7159 (2)	0.19942 (14)	0.0507 (8)	
N10	0.5106 (3)	0.77723 (19)	0.31263 (13)	0.0432 (7)	
N11	0.5722 (3)	0.9187 (2)	0.36334 (15)	0.0597 (9)	
H11	0.615093	0.965062	0.392887	0.072*	
N12	0.4736 (4)	0.9271 (2)	0.30800 (16)	0.0621 (9)	
N13	0.6489 (3)	0.58921 (19)	0.31364 (13)	0.0455 (7)	
N14	0.6070 (3)	0.65457 (19)	0.19219 (13)	0.0445 (7)	
N15	0.7518 (3)	0.6810 (2)	0.12811 (14)	0.0543 (8)	
H15	0.783958	0.695836	0.093650	0.065*	
N16	0.8289 (3)	0.6538 (2)	0.18426 (14)	0.0529 (8)	
C1	0.2527 (4)	0.5063 (2)	0.28770 (16)	0.0386 (8)	
C2	0.2758 (3)	0.4641 (2)	0.21719 (16)	0.0374 (8)	
C3	−0.1234 (4)	0.4204 (3)	0.12807 (18)	0.0589 (11)	
H3A	−0.043788	0.429579	0.110984	0.071*	
C4	−0.2462 (5)	0.4407 (3)	0.0907 (2)	0.0667 (12)	
H4	−0.249256	0.462694	0.048877	0.080*	
C5	−0.3627 (5)	0.4285 (3)	0.1156 (2)	0.0702 (13)	
H5	−0.446072	0.441986	0.090806	0.084*	
C6	−0.3568 (4)	0.3958 (3)	0.17799 (19)	0.0575 (10)	
H6	−0.435366	0.387506	0.196046	0.069*	
C7	−0.2312 (4)	0.3760 (2)	0.21249 (16)	0.0427 (8)	
C8	−0.2112 (4)	0.3405 (2)	0.27900 (16)	0.0421 (8)	
C9	−0.1053 (4)	0.2887 (3)	0.36643 (17)	0.0503 (9)	
C10	−0.0023 (4)	0.2548 (4)	0.4192 (2)	0.0807 (14)	
H10A	0.024759	0.197175	0.400311	0.121*	
H10B	−0.042144	0.244484	0.457843	0.121*	
H10C	0.077127	0.300738	0.433744	0.121*	
C11	0.3084 (4)	0.2830 (3)	0.36143 (18)	0.0577 (10)	
H11A	0.326791	0.347058	0.375872	0.069*	
C12	0.3799 (4)	0.2239 (3)	0.3996 (2)	0.0659 (12)	
H12	0.446270	0.247802	0.438745	0.079*	
C13	0.3528 (4)	0.1295 (3)	0.3796 (2)	0.0704 (13)	
H13	0.398179	0.088386	0.405802	0.084*	
C14	0.2570 (4)	0.0956 (3)	0.31978 (18)	0.0625 (11)	
H14	0.237582	0.031753	0.304686	0.075*	
C15	0.1912 (4)	0.1594 (3)	0.28328 (17)	0.0463 (9)	
C16	0.0893 (4)	0.1317 (3)	0.21895 (17)	0.0491 (9)	
C17	−0.0606 (4)	0.1457 (3)	0.13178 (19)	0.0617 (11)	
C18	−0.1622 (5)	0.1779 (3)	0.0775 (2)	0.0832 (15)	
H18A	−0.235851	0.200596	0.096682	0.125*	
H18B	−0.198935	0.126680	0.041786	0.125*	
H18C	−0.117722	0.227346	0.058849	0.125*	
C19	0.2082 (4)	0.6836 (3)	0.14435 (19)	0.0642 (11)	
H19	0.188657	0.619209	0.131749	0.077*	
C20	0.1332 (4)	0.7406 (4)	0.1055 (2)	0.0755 (14)	
H20	0.063650	0.715303	0.067988	0.091*	
C21	0.1627 (5)	0.8348 (4)	0.1230 (2)	0.0803 (15)	
H21	0.114664	0.874722	0.096693	0.096*	
C22	0.2640 (5)	0.8712 (3)	0.17987 (19)	0.0702 (13)	
H22	0.284770	0.935471	0.192785	0.084*	
C23	0.3334 (4)	0.8092 (3)	0.21697 (17)	0.0501 (9)	
C24	0.4396 (4)	0.8401 (3)	0.27894 (17)	0.0485 (9)	
C25	0.5944 (4)	0.8301 (3)	0.36629 (17)	0.0487 (9)	
C26	0.6950 (4)	0.8004 (3)	0.42185 (18)	0.0677 (12)	
H26A	0.650605	0.751460	0.440960	0.102*	
H26B	0.729815	0.852738	0.456919	0.102*	
H26C	0.769906	0.777834	0.403759	0.102*	
C27	0.6645 (4)	0.5565 (3)	0.37394 (18)	0.0592 (11)	
H27	0.586820	0.544259	0.392017	0.071*	
C28	0.7898 (5)	0.5401 (3)	0.4102 (2)	0.0684 (12)	
H28	0.796418	0.518366	0.452334	0.082*	
C29	0.9047 (5)	0.5560 (3)	0.3841 (2)	0.0702 (12)	
H29	0.990504	0.545042	0.407988	0.084*	
C30	0.8916 (4)	0.5886 (3)	0.32143 (19)	0.0594 (11)	
H30	0.968095	0.599935	0.302321	0.071*	
C31	0.7626 (4)	0.6039 (2)	0.28800 (16)	0.0436 (8)	
C32	0.7366 (4)	0.6382 (2)	0.22115 (16)	0.0429 (8)	
C33	0.6205 (4)	0.6820 (3)	0.13259 (17)	0.0515 (10)	
C34	0.5110 (4)	0.7077 (3)	0.07895 (18)	0.0773 (14)	
H34A	0.486349	0.768028	0.093915	0.116*	
H34B	0.431515	0.661919	0.070729	0.116*	
H34C	0.544165	0.709851	0.037429	0.116*	
S1	0.19815 (11)	0.15158 (8)	−0.01003 (5)	0.0625 (3)	
O5	0.1921 (3)	0.25052 (19)	−0.00942 (11)	0.0669 (8)	
O6	0.2457 (4)	0.1123 (2)	−0.06992 (14)	0.1116 (13)	
O7	0.0732 (4)	0.1004 (3)	−0.0048 (2)	0.1409 (18)	
C35	0.3234 (4)	0.1378 (3)	0.06207 (17)	0.0470 (9)	
C36	0.3414 (4)	0.1958 (3)	0.12319 (18)	0.0534 (10)	
H36	0.287580	0.244558	0.126915	0.064*	
C37	0.4393 (4)	0.1816 (3)	0.17913 (18)	0.0566 (10)	
H37	0.450062	0.221047	0.220260	0.068*	
C38	0.5207 (4)	0.1109 (3)	0.1753 (2)	0.0596 (11)	
C39	0.5008 (5)	0.0528 (3)	0.1146 (2)	0.0840 (15)	
H39	0.554298	0.003751	0.111059	0.101*	
C40	0.4026 (5)	0.0656 (3)	0.0582 (2)	0.0755 (13)	
H40	0.390360	0.024997	0.017557	0.091*	
C41	0.6316 (5)	0.0972 (3)	0.2360 (2)	0.0912 (16)	
H41A	0.718786	0.127442	0.231879	0.137*	
H41B	0.637337	0.031548	0.236750	0.137*	
H41C	0.608687	0.123984	0.277673	0.137*	
S2	0.30295 (10)	0.86078 (7)	0.49751 (4)	0.0531 (3)	
O8	0.4317 (3)	0.8827 (3)	0.47784 (15)	0.0979 (12)	
O9	0.2802 (3)	0.9233 (2)	0.55456 (13)	0.0826 (9)	
O10	0.2837 (3)	0.76442 (18)	0.51006 (11)	0.0620 (7)	
C42	0.1701 (4)	0.8707 (2)	0.42682 (17)	0.0451 (9)	
C43	0.0828 (4)	0.9383 (3)	0.4305 (2)	0.0641 (11)	
H43	0.092198	0.979305	0.470797	0.077*	
C44	−0.0196 (4)	0.9454 (3)	0.3740 (2)	0.0705 (12)	
H44	−0.078646	0.991101	0.376928	0.085*	
C45	−0.0353 (4)	0.8857 (3)	0.3134 (2)	0.0570 (10)	
C46	0.0549 (4)	0.8200 (3)	0.31052 (18)	0.0561 (10)	
H46	0.047203	0.779862	0.269950	0.067*	
C47	0.1565 (4)	0.8119 (3)	0.36615 (17)	0.0529 (10)	
H47	0.216238	0.766700	0.362861	0.063*	
C48	−0.1485 (5)	0.8935 (3)	0.2527 (2)	0.0843 (15)	
H48A	−0.121972	0.869788	0.211156	0.126*	
H48B	−0.163294	0.957970	0.251877	0.126*	
H48C	−0.232041	0.857815	0.256327	0.126*	
O1W	−0.018 (2)	0.4596 (12)	−0.0131 (10)	0.159 (7)*	0.25
H1WA	−0.026571	0.516586	−0.000700	0.239*	0.25
H1WB	0.065089	0.453836	0.003990	0.239*	0.25
O2W	0.2693 (5)	0.4417 (2)	0.04606 (14)	0.1319 (16)	
H2WA	0.302346	0.458579	0.088428	0.198*	
H2WB	0.249386	0.382599	0.033148	0.198*	
O4W	0.2661 (6)	0.5760 (3)	0.45735 (18)	0.1135 (19)	0.75
H4WA	0.264626	0.557925	0.414491	0.170*	0.75
H4WB	0.272806	0.635525	0.470971	0.170*	0.75
O5W	0.4606 (9)	0.4449 (5)	0.4825 (4)	0.136 (3)*	0.5
H5WA	0.536786	0.439800	0.510473	0.205*	0.5
H5WB	0.458166	0.499590	0.469693	0.205*	0.5
O3W	0.3943 (15)	0.6210 (10)	0.4573 (6)	0.094 (4)*	0.25
H3WA	0.376336	0.611397	0.413001	0.141*	0.25
H3WB	0.362126	0.671997	0.469671	0.141*	0.25

### Atomic displacement parameters (Å^2^)

**Table:**

	*U*^11^	*U*^22^	*U*^33^	*U*^12^	*U*^13^	*U*^23^
Fe1	0.0381 (3)	0.0391 (3)	0.0426 (3)	−0.0045 (2)	0.0063 (2)	0.0040 (2)
Fe2	0.0400 (3)	0.0413 (3)	0.0438 (3)	−0.0073 (2)	0.0079 (2)	0.0018 (2)
O1	0.0493 (16)	0.0481 (15)	0.0453 (13)	−0.0074 (12)	0.0213 (12)	−0.0037 (11)
O2	0.0551 (17)	0.0440 (15)	0.0427 (13)	−0.0099 (13)	0.0163 (12)	−0.0072 (11)
O3	0.0580 (17)	0.0450 (15)	0.0430 (13)	−0.0135 (13)	0.0169 (12)	−0.0121 (12)
O4	0.0513 (16)	0.0495 (15)	0.0445 (13)	−0.0114 (13)	0.0222 (12)	−0.0044 (12)
N1	0.052 (2)	0.0367 (17)	0.0414 (16)	0.0027 (15)	0.0042 (14)	0.0044 (13)
N2	0.0431 (19)	0.0498 (19)	0.0416 (15)	−0.0004 (15)	0.0066 (13)	0.0113 (14)
N3	0.052 (2)	0.070 (2)	0.0449 (17)	−0.0014 (18)	0.0152 (15)	0.0103 (16)
N4	0.050 (2)	0.060 (2)	0.0526 (18)	0.0041 (17)	0.0121 (16)	0.0090 (16)
N5	0.0345 (18)	0.053 (2)	0.0460 (16)	−0.0007 (15)	0.0018 (13)	0.0039 (15)
N6	0.0462 (19)	0.0392 (17)	0.0406 (15)	−0.0001 (14)	−0.0013 (13)	0.0033 (14)
N7	0.129 (4)	0.044 (2)	0.071 (2)	0.015 (2)	−0.045 (2)	−0.0138 (18)
N8	0.115 (3)	0.047 (2)	0.070 (2)	0.020 (2)	−0.031 (2)	−0.0039 (18)
N9	0.0393 (19)	0.058 (2)	0.0488 (17)	−0.0012 (16)	0.0014 (14)	0.0024 (16)
N10	0.0425 (18)	0.0401 (17)	0.0431 (16)	−0.0029 (14)	0.0056 (14)	0.0018 (14)
N11	0.073 (3)	0.046 (2)	0.0486 (18)	−0.0023 (18)	−0.0015 (17)	−0.0093 (15)
N12	0.073 (3)	0.049 (2)	0.0565 (19)	0.0093 (19)	0.0003 (18)	−0.0017 (17)
N13	0.055 (2)	0.0399 (17)	0.0403 (16)	−0.0028 (15)	0.0102 (14)	0.0066 (14)
N14	0.0420 (19)	0.0462 (18)	0.0436 (16)	−0.0027 (15)	0.0077 (14)	0.0076 (14)
N15	0.053 (2)	0.068 (2)	0.0427 (17)	−0.0013 (18)	0.0145 (15)	0.0130 (16)
N16	0.050 (2)	0.057 (2)	0.0520 (18)	0.0007 (16)	0.0143 (16)	0.0084 (16)
C1	0.038 (2)	0.038 (2)	0.0391 (18)	0.0007 (17)	0.0105 (16)	0.0000 (16)
C2	0.037 (2)	0.036 (2)	0.0373 (18)	0.0023 (17)	0.0074 (15)	−0.0002 (16)
C3	0.074 (3)	0.053 (3)	0.049 (2)	0.000 (2)	0.010 (2)	0.013 (2)
C4	0.092 (4)	0.048 (3)	0.053 (2)	0.008 (3)	−0.006 (2)	0.014 (2)
C5	0.072 (3)	0.059 (3)	0.069 (3)	0.014 (3)	−0.012 (2)	0.012 (2)
C6	0.048 (3)	0.053 (3)	0.066 (2)	0.009 (2)	−0.001 (2)	0.004 (2)
C7	0.046 (2)	0.034 (2)	0.0463 (19)	0.0050 (17)	0.0077 (17)	0.0026 (16)
C8	0.043 (2)	0.036 (2)	0.0445 (19)	0.0007 (17)	0.0064 (17)	0.0014 (16)
C9	0.048 (3)	0.057 (2)	0.043 (2)	−0.003 (2)	0.0067 (18)	0.0087 (18)
C10	0.067 (3)	0.119 (4)	0.062 (3)	0.008 (3)	0.012 (2)	0.038 (3)
C11	0.044 (2)	0.062 (3)	0.057 (2)	0.001 (2)	−0.0035 (19)	0.000 (2)
C12	0.046 (3)	0.086 (3)	0.056 (2)	0.008 (2)	−0.0080 (19)	0.001 (2)
C13	0.066 (3)	0.088 (4)	0.057 (2)	0.029 (3)	0.000 (2)	0.014 (3)
C14	0.070 (3)	0.057 (3)	0.055 (2)	0.021 (2)	0.000 (2)	0.001 (2)
C15	0.043 (2)	0.049 (2)	0.0449 (19)	0.0076 (19)	0.0052 (17)	0.0060 (18)
C16	0.052 (2)	0.040 (2)	0.050 (2)	0.0036 (19)	0.0015 (18)	0.0043 (18)
C17	0.075 (3)	0.044 (2)	0.053 (2)	0.001 (2)	−0.012 (2)	0.0018 (19)
C18	0.099 (4)	0.061 (3)	0.066 (3)	0.010 (3)	−0.032 (2)	−0.005 (2)
C19	0.045 (3)	0.074 (3)	0.062 (2)	−0.003 (2)	−0.002 (2)	−0.004 (2)
C20	0.049 (3)	0.106 (4)	0.060 (3)	0.015 (3)	−0.011 (2)	−0.001 (3)
C21	0.080 (4)	0.098 (4)	0.061 (3)	0.041 (3)	−0.002 (2)	0.009 (3)
C22	0.076 (3)	0.072 (3)	0.059 (2)	0.029 (3)	0.000 (2)	0.004 (2)
C23	0.047 (2)	0.056 (3)	0.046 (2)	0.007 (2)	0.0083 (17)	0.0035 (19)
C24	0.051 (2)	0.047 (2)	0.044 (2)	0.004 (2)	0.0057 (17)	0.0004 (18)
C25	0.051 (2)	0.044 (2)	0.047 (2)	−0.0008 (19)	0.0079 (18)	0.0006 (18)
C26	0.075 (3)	0.062 (3)	0.052 (2)	0.002 (2)	−0.011 (2)	−0.002 (2)
C27	0.075 (3)	0.053 (3)	0.052 (2)	0.004 (2)	0.017 (2)	0.014 (2)
C28	0.095 (4)	0.059 (3)	0.047 (2)	0.010 (3)	0.000 (2)	0.016 (2)
C29	0.068 (3)	0.068 (3)	0.067 (3)	0.012 (3)	−0.007 (2)	0.015 (2)
C30	0.051 (3)	0.062 (3)	0.062 (2)	0.005 (2)	0.006 (2)	0.009 (2)
C31	0.048 (2)	0.034 (2)	0.0438 (19)	−0.0022 (17)	0.0052 (17)	0.0004 (16)
C32	0.043 (2)	0.038 (2)	0.0451 (19)	−0.0010 (17)	0.0081 (17)	0.0019 (16)
C33	0.052 (3)	0.057 (2)	0.044 (2)	−0.002 (2)	0.0097 (18)	0.0089 (19)
C34	0.058 (3)	0.120 (4)	0.053 (2)	0.000 (3)	0.005 (2)	0.029 (3)
S1	0.0572 (7)	0.0586 (7)	0.0618 (6)	−0.0095 (5)	−0.0082 (5)	0.0159 (6)
O5	0.089 (2)	0.0629 (19)	0.0442 (14)	0.0177 (16)	0.0011 (14)	0.0063 (13)
O6	0.165 (4)	0.101 (3)	0.0500 (17)	0.034 (3)	−0.011 (2)	−0.0208 (17)
O7	0.061 (2)	0.183 (4)	0.164 (3)	−0.046 (2)	−0.036 (2)	0.115 (3)
C35	0.044 (2)	0.043 (2)	0.053 (2)	0.0010 (18)	0.0101 (17)	0.0041 (18)
C36	0.049 (2)	0.047 (2)	0.059 (2)	0.0110 (19)	0.0022 (19)	−0.0021 (19)
C37	0.058 (3)	0.056 (3)	0.049 (2)	0.000 (2)	0.0037 (19)	−0.0006 (19)
C38	0.057 (3)	0.048 (2)	0.068 (3)	0.000 (2)	−0.004 (2)	0.016 (2)
C39	0.081 (4)	0.065 (3)	0.102 (4)	0.038 (3)	0.001 (3)	0.004 (3)
C40	0.081 (4)	0.069 (3)	0.068 (3)	0.023 (3)	0.006 (2)	−0.017 (2)
C41	0.087 (4)	0.066 (3)	0.106 (4)	0.008 (3)	−0.022 (3)	0.029 (3)
S2	0.0474 (6)	0.0611 (7)	0.0441 (5)	−0.0079 (5)	0.0009 (4)	0.0066 (5)
O8	0.0445 (19)	0.161 (3)	0.086 (2)	−0.019 (2)	0.0025 (16)	0.053 (2)
O9	0.098 (3)	0.074 (2)	0.0562 (16)	0.0014 (18)	−0.0095 (16)	−0.0212 (15)
O10	0.081 (2)	0.0599 (18)	0.0435 (14)	0.0076 (15)	0.0072 (13)	0.0104 (13)
C42	0.043 (2)	0.042 (2)	0.049 (2)	−0.0022 (18)	0.0102 (17)	0.0060 (17)
C43	0.063 (3)	0.064 (3)	0.061 (2)	0.007 (2)	0.011 (2)	−0.009 (2)
C44	0.061 (3)	0.057 (3)	0.091 (3)	0.020 (2)	0.007 (3)	0.005 (3)
C45	0.053 (3)	0.051 (2)	0.062 (2)	−0.001 (2)	0.002 (2)	0.013 (2)
C46	0.058 (3)	0.059 (3)	0.045 (2)	0.005 (2)	0.0049 (19)	−0.0040 (19)
C47	0.052 (3)	0.052 (2)	0.051 (2)	0.012 (2)	0.0035 (18)	−0.0016 (19)
C48	0.074 (3)	0.082 (4)	0.088 (3)	0.012 (3)	−0.012 (3)	0.023 (3)
O2W	0.252 (5)	0.086 (3)	0.0506 (18)	0.024 (3)	0.012 (2)	0.0135 (18)
O4W	0.201 (6)	0.096 (4)	0.051 (2)	0.046 (4)	0.034 (3)	0.004 (2)

### Geometric parameters (Å, º)

**Table:**

Fe1—O1	2.171 (2)	C18—H18C	0.9600
Fe1—O2	2.123 (2)	C19—H19	0.9300
Fe1—N1	2.203 (3)	C19—C20	1.372 (5)
Fe1—N2	2.150 (3)	C20—H20	0.9300
Fe1—N5	2.197 (3)	C20—C21	1.359 (6)
Fe1—N6	2.162 (3)	C21—H21	0.9300
Fe2—O3	2.123 (2)	C21—C22	1.381 (6)
Fe2—O4	2.157 (2)	C22—H22	0.9300
Fe2—N9	2.209 (3)	C22—C23	1.381 (5)
Fe2—N10	2.165 (3)	C23—C24	1.460 (5)
Fe2—N13	2.206 (3)	C25—C26	1.478 (5)
Fe2—N14	2.159 (3)	C26—H26A	0.9600
O1—C1	1.241 (4)	C26—H26B	0.9600
O2—C2	1.243 (4)	C26—H26C	0.9600
O3—C1	1.245 (4)	C27—H27	0.9300
O4—C2	1.249 (4)	C27—C28	1.371 (5)
N1—C3	1.349 (4)	C28—H28	0.9300
N1—C7	1.343 (4)	C28—C29	1.364 (6)
N2—C8	1.363 (4)	C29—H29	0.9300
N2—C9	1.333 (4)	C29—C30	1.384 (5)
N3—H3	0.8600	C30—H30	0.9300
N3—N4	1.350 (4)	C30—C31	1.377 (5)
N3—C9	1.332 (4)	C31—C32	1.471 (4)
N4—C8	1.320 (4)	C33—C34	1.481 (5)
N5—C11	1.344 (4)	C34—H34A	0.9600
N5—C15	1.344 (4)	C34—H34B	0.9600
N6—C16	1.364 (4)	C34—H34C	0.9600
N6—C17	1.323 (4)	S1—O5	1.439 (3)
N7—H7	0.8600	S1—O6	1.454 (3)
N7—N8	1.356 (4)	S1—O7	1.419 (3)
N7—C17	1.318 (5)	S1—C35	1.758 (4)
N8—C16	1.305 (4)	C35—C36	1.375 (5)
N9—C19	1.338 (4)	C35—C40	1.371 (5)
N9—C23	1.344 (4)	C36—H36	0.9300
N10—C24	1.365 (4)	C36—C37	1.383 (5)
N10—C25	1.334 (4)	C37—H37	0.9300
N11—H11	0.8600	C37—C38	1.368 (5)
N11—N12	1.355 (4)	C38—C39	1.368 (6)
N11—C25	1.329 (4)	C38—C41	1.520 (5)
N12—C24	1.309 (4)	C39—H39	0.9300
N13—C27	1.339 (4)	C39—C40	1.384 (5)
N13—C31	1.345 (4)	C40—H40	0.9300
N14—C32	1.361 (4)	C41—H41A	0.9600
N14—C33	1.334 (4)	C41—H41B	0.9600
N15—H15	0.8600	C41—H41C	0.9600
N15—N16	1.354 (4)	S2—O8	1.435 (3)
N15—C33	1.331 (4)	S2—O9	1.444 (3)
N16—C32	1.312 (4)	S2—O10	1.450 (3)
C1—C2	1.546 (4)	S2—C42	1.768 (4)
C3—H3A	0.9300	C42—C43	1.373 (5)
C3—C4	1.379 (5)	C42—C47	1.379 (5)
C4—H4	0.9300	C43—H43	0.9300
C4—C5	1.357 (6)	C43—C44	1.388 (5)
C5—H5	0.9300	C44—H44	0.9300
C5—C6	1.385 (5)	C44—C45	1.381 (5)
C6—H6	0.9300	C45—C46	1.372 (5)
C6—C7	1.377 (5)	C45—C48	1.512 (5)
C7—C8	1.472 (4)	C46—H46	0.9300
C9—C10	1.484 (5)	C46—C47	1.375 (5)
C10—H10A	0.9600	C47—H47	0.9300
C10—H10B	0.9600	C48—H48A	0.9600
C10—H10C	0.9600	C48—H48B	0.9600
C11—H11A	0.9300	C48—H48C	0.9600
C11—C12	1.370 (5)	O1W—H1WA	0.8500
C12—H12	0.9300	O1W—H1WB	0.8499
C12—C13	1.366 (6)	O2W—H2WA	0.8482
C13—H13	0.9300	O2W—H2WB	0.8577
C13—C14	1.384 (5)	O4W—H4WA	0.8679
C14—H14	0.9300	O4W—H4WB	0.8665
C14—C15	1.378 (5)	O5W—H5WA	0.8651
C15—C16	1.465 (5)	O5W—H5WB	0.8618
C17—C18	1.483 (5)	O3W—H3WA	0.8642
C18—H18A	0.9600	O3W—H3WB	0.8617
C18—H18B	0.9600		
			
O1—Fe1—N1	98.95 (10)	N7—C17—C18	123.1 (4)
O1—Fe1—N5	91.45 (10)	C17—C18—H18A	109.5
O2—Fe1—O1	76.88 (8)	C17—C18—H18B	109.5
O2—Fe1—N1	94.10 (10)	C17—C18—H18C	109.5
O2—Fe1—N2	163.77 (10)	H18A—C18—H18B	109.5
O2—Fe1—N5	96.26 (10)	H18A—C18—H18C	109.5
O2—Fe1—N6	97.91 (10)	H18B—C18—H18C	109.5
N2—Fe1—O1	91.45 (10)	N9—C19—H19	118.3
N2—Fe1—N1	76.39 (10)	N9—C19—C20	123.4 (4)
N2—Fe1—N5	95.26 (10)	C20—C19—H19	118.3
N2—Fe1—N6	95.76 (11)	C19—C20—H20	120.7
N5—Fe1—N1	166.75 (11)	C21—C20—C19	118.6 (4)
N6—Fe1—O1	167.01 (10)	C21—C20—H20	120.7
N6—Fe1—N1	93.25 (10)	C20—C21—H21	120.0
N6—Fe1—N5	77.17 (10)	C20—C21—C22	119.9 (4)
O3—Fe2—O4	77.01 (8)	C22—C21—H21	120.0
O3—Fe2—N9	95.46 (10)	C21—C22—H22	121.0
O3—Fe2—N10	99.08 (9)	C21—C22—C23	118.0 (4)
O3—Fe2—N13	96.09 (10)	C23—C22—H22	121.0
O3—Fe2—N14	164.19 (10)	N9—C23—C22	122.9 (4)
O4—Fe2—N9	90.81 (10)	N9—C23—C24	114.7 (3)
O4—Fe2—N10	166.87 (10)	C22—C23—C24	122.4 (4)
O4—Fe2—N13	100.13 (10)	N10—C24—C23	120.9 (3)
O4—Fe2—N14	90.65 (9)	N12—C24—N10	114.2 (3)
N10—Fe2—N9	76.99 (11)	N12—C24—C23	124.9 (3)
N10—Fe2—N13	92.72 (10)	N10—C25—C26	128.6 (3)
N13—Fe2—N9	165.61 (11)	N11—C25—N10	108.2 (3)
N14—Fe2—N9	94.55 (10)	N11—C25—C26	123.2 (3)
N14—Fe2—N10	95.07 (10)	C25—C26—H26A	109.5
N14—Fe2—N13	76.15 (10)	C25—C26—H26B	109.5
C1—O1—Fe1	113.9 (2)	C25—C26—H26C	109.5
C2—O2—Fe1	115.3 (2)	H26A—C26—H26B	109.5
C1—O3—Fe2	115.4 (2)	H26A—C26—H26C	109.5
C2—O4—Fe2	114.1 (2)	H26B—C26—H26C	109.5
C3—N1—Fe1	126.0 (3)	N13—C27—H27	118.5
C7—N1—Fe1	115.9 (2)	N13—C27—C28	123.0 (4)
C7—N1—C3	117.7 (3)	C28—C27—H27	118.5
C8—N2—Fe1	113.6 (2)	C27—C28—H28	120.3
C9—N2—Fe1	142.8 (2)	C29—C28—C27	119.5 (4)
C9—N2—C8	103.6 (3)	C29—C28—H28	120.3
N4—N3—H3	124.0	C28—C29—H29	120.6
C9—N3—H3	124.0	C28—C29—C30	118.9 (4)
C9—N3—N4	112.1 (3)	C30—C29—H29	120.6
C8—N4—N3	101.7 (3)	C29—C30—H30	120.8
C11—N5—Fe1	127.1 (3)	C31—C30—C29	118.5 (4)
C15—N5—Fe1	115.0 (2)	C31—C30—H30	120.8
C15—N5—C11	117.3 (3)	N13—C31—C30	123.1 (3)
C16—N6—Fe1	111.9 (2)	N13—C31—C32	113.9 (3)
C17—N6—Fe1	144.8 (2)	C30—C31—C32	123.1 (3)
C17—N6—C16	103.3 (3)	N14—C32—C31	119.8 (3)
N8—N7—H7	124.2	N16—C32—N14	114.6 (3)
C17—N7—H7	124.2	N16—C32—C31	125.6 (3)
C17—N7—N8	111.6 (3)	N14—C33—C34	127.4 (4)
C16—N8—N7	101.8 (3)	N15—C33—N14	108.0 (3)
C19—N9—Fe2	128.0 (3)	N15—C33—C34	124.6 (3)
C19—N9—C23	117.1 (3)	C33—C34—H34A	109.5
C23—N9—Fe2	114.2 (2)	C33—C34—H34B	109.5
C24—N10—Fe2	111.8 (2)	C33—C34—H34C	109.5
C25—N10—Fe2	144.1 (2)	H34A—C34—H34B	109.5
C25—N10—C24	103.8 (3)	H34A—C34—H34C	109.5
N12—N11—H11	124.2	H34B—C34—H34C	109.5
C25—N11—H11	124.2	O5—S1—O6	110.60 (19)
C25—N11—N12	111.6 (3)	O5—S1—C35	106.93 (17)
C24—N12—N11	102.2 (3)	O6—S1—C35	106.78 (19)
C27—N13—Fe2	126.7 (3)	O7—S1—O5	114.3 (2)
C27—N13—C31	117.1 (3)	O7—S1—O6	110.8 (3)
C31—N13—Fe2	115.8 (2)	O7—S1—C35	106.96 (18)
C32—N14—Fe2	113.6 (2)	C36—C35—S1	121.8 (3)
C33—N14—Fe2	142.5 (3)	C40—C35—S1	119.4 (3)
C33—N14—C32	103.7 (3)	C40—C35—C36	118.8 (3)
N16—N15—H15	124.0	C35—C36—H36	120.0
C33—N15—H15	124.0	C35—C36—C37	120.1 (3)
C33—N15—N16	112.0 (3)	C37—C36—H36	120.0
C32—N16—N15	101.7 (3)	C36—C37—H37	119.3
O1—C1—O3	126.5 (3)	C38—C37—C36	121.4 (4)
O1—C1—C2	116.8 (3)	C38—C37—H37	119.3
O3—C1—C2	116.6 (3)	C37—C38—C39	118.1 (4)
O2—C2—O4	126.3 (3)	C37—C38—C41	121.5 (4)
O2—C2—C1	117.0 (3)	C39—C38—C41	120.4 (4)
O4—C2—C1	116.7 (3)	C38—C39—H39	119.4
N1—C3—H3A	119.0	C38—C39—C40	121.2 (4)
N1—C3—C4	122.1 (4)	C40—C39—H39	119.4
C4—C3—H3A	119.0	C35—C40—C39	120.4 (4)
C3—C4—H4	120.3	C35—C40—H40	119.8
C5—C4—C3	119.4 (4)	C39—C40—H40	119.8
C5—C4—H4	120.3	C38—C41—H41A	109.5
C4—C5—H5	120.1	C38—C41—H41B	109.5
C4—C5—C6	119.8 (4)	C38—C41—H41C	109.5
C6—C5—H5	120.1	H41A—C41—H41B	109.5
C5—C6—H6	121.0	H41A—C41—H41C	109.5
C7—C6—C5	118.0 (4)	H41B—C41—H41C	109.5
C7—C6—H6	121.0	O8—S2—O9	114.8 (2)
N1—C7—C6	123.1 (3)	O8—S2—O10	111.9 (2)
N1—C7—C8	113.6 (3)	O8—S2—C42	107.17 (16)
C6—C7—C8	123.4 (3)	O9—S2—O10	110.33 (16)
N2—C8—C7	120.0 (3)	O9—S2—C42	106.35 (17)
N4—C8—N2	114.5 (3)	O10—S2—C42	105.76 (17)
N4—C8—C7	125.5 (3)	C43—C42—S2	121.0 (3)
N2—C9—C10	127.2 (3)	C43—C42—C47	119.2 (3)
N3—C9—N2	108.2 (3)	C47—C42—S2	119.7 (3)
N3—C9—C10	124.5 (3)	C42—C43—H43	120.1
C9—C10—H10A	109.5	C42—C43—C44	119.9 (4)
C9—C10—H10B	109.5	C44—C43—H43	120.1
C9—C10—H10C	109.5	C43—C44—H44	119.4
H10A—C10—H10B	109.5	C45—C44—C43	121.2 (4)
H10A—C10—H10C	109.5	C45—C44—H44	119.4
H10B—C10—H10C	109.5	C44—C45—C48	120.5 (4)
N5—C11—H11A	118.7	C46—C45—C44	117.9 (4)
N5—C11—C12	122.7 (4)	C46—C45—C48	121.6 (4)
C12—C11—H11A	118.7	C45—C46—H46	119.2
C11—C12—H12	120.3	C45—C46—C47	121.6 (4)
C13—C12—C11	119.4 (4)	C47—C46—H46	119.2
C13—C12—H12	120.3	C42—C47—H47	119.9
C12—C13—H13	120.4	C46—C47—C42	120.3 (3)
C12—C13—C14	119.2 (4)	C46—C47—H47	119.9
C14—C13—H13	120.4	C45—C48—H48A	109.5
C13—C14—H14	120.9	C45—C48—H48B	109.5
C15—C14—C13	118.1 (4)	C45—C48—H48C	109.5
C15—C14—H14	120.9	H48A—C48—H48B	109.5
N5—C15—C14	123.2 (3)	H48A—C48—H48C	109.5
N5—C15—C16	114.0 (3)	H48B—C48—H48C	109.5
C14—C15—C16	122.8 (3)	H1WA—O1W—H1WB	104.5
N6—C16—C15	121.1 (3)	H2WA—O2W—H2WB	116.3
N8—C16—N6	114.5 (3)	H4WA—O4W—H4WB	117.9
N8—C16—C15	124.4 (3)	H5WA—O5W—H5WB	112.9
N6—C17—C18	128.1 (4)	H3WA—O3W—H3WB	107.7
N7—C17—N6	108.8 (3)		
			
Fe1—O1—C1—O3	−178.1 (3)	C8—N2—C9—C10	−180.0 (4)
Fe1—O1—C1—C2	1.9 (4)	C9—N2—C8—N4	−0.7 (4)
Fe1—O2—C2—O4	178.1 (3)	C9—N2—C8—C7	179.9 (3)
Fe1—O2—C2—C1	−2.0 (4)	C9—N3—N4—C8	−0.3 (4)
Fe1—N1—C3—C4	171.0 (3)	C11—N5—C15—C14	3.3 (5)
Fe1—N1—C7—C6	−172.4 (3)	C11—N5—C15—C16	−177.8 (3)
Fe1—N1—C7—C8	7.7 (4)	C11—C12—C13—C14	2.2 (6)
Fe1—N2—C8—N4	177.6 (2)	C12—C13—C14—C15	−0.9 (6)
Fe1—N2—C8—C7	−1.8 (4)	C13—C14—C15—N5	−2.0 (6)
Fe1—N2—C9—N3	−176.9 (3)	C13—C14—C15—C16	179.2 (4)
Fe1—N2—C9—C10	2.6 (7)	C14—C15—C16—N6	172.9 (3)
Fe1—N5—C11—C12	169.2 (3)	C14—C15—C16—N8	−8.9 (6)
Fe1—N5—C15—C14	−168.8 (3)	C15—N5—C11—C12	−1.9 (5)
Fe1—N5—C15—C16	10.1 (4)	C16—N6—C17—N7	0.1 (5)
Fe1—N6—C16—N8	−179.6 (3)	C16—N6—C17—C18	−180.0 (4)
Fe1—N6—C16—C15	−1.2 (4)	C17—N6—C16—N8	−0.1 (5)
Fe1—N6—C17—N7	179.3 (3)	C17—N6—C16—C15	178.3 (4)
Fe1—N6—C17—C18	−0.8 (8)	C17—N7—N8—C16	−0.1 (5)
Fe2—O3—C1—O1	−177.3 (3)	C19—N9—C23—C22	−1.8 (5)
Fe2—O3—C1—C2	2.7 (4)	C19—N9—C23—C24	177.7 (3)
Fe2—O4—C2—O2	177.4 (3)	C19—C20—C21—C22	−1.6 (7)
Fe2—O4—C2—C1	−2.5 (3)	C20—C21—C22—C23	0.6 (7)
Fe2—N9—C19—C20	−168.9 (3)	C21—C22—C23—N9	1.1 (6)
Fe2—N9—C23—C22	169.3 (3)	C21—C22—C23—C24	−178.3 (4)
Fe2—N9—C23—C24	−11.2 (4)	C22—C23—C24—N10	−177.0 (3)
Fe2—N10—C24—N12	−175.3 (3)	C22—C23—C24—N12	4.6 (6)
Fe2—N10—C24—C23	6.1 (4)	C23—N9—C19—C20	0.7 (6)
Fe2—N10—C25—N11	172.5 (3)	C24—N10—C25—N11	0.0 (4)
Fe2—N10—C25—C26	−8.4 (7)	C24—N10—C25—C26	179.1 (4)
Fe2—N13—C27—C28	−170.8 (3)	C25—N10—C24—N12	−0.1 (4)
Fe2—N13—C31—C30	172.0 (3)	C25—N10—C24—C23	−178.6 (3)
Fe2—N13—C31—C32	−7.7 (4)	C25—N11—N12—C24	−0.1 (4)
Fe2—N14—C32—N16	−176.2 (2)	C27—N13—C31—C30	−1.4 (5)
Fe2—N14—C32—C31	5.1 (4)	C27—N13—C31—C32	179.0 (3)
Fe2—N14—C33—N15	174.8 (3)	C27—C28—C29—C30	0.2 (7)
Fe2—N14—C33—C34	−6.4 (7)	C28—C29—C30—C31	0.1 (6)
O1—C1—C2—O2	0.0 (4)	C29—C30—C31—N13	0.5 (6)
O1—C1—C2—O4	179.9 (3)	C29—C30—C31—C32	−179.9 (3)
O3—C1—C2—O2	180.0 (3)	C30—C31—C32—N14	−177.9 (3)
O3—C1—C2—O4	−0.1 (4)	C30—C31—C32—N16	3.5 (6)
N1—C3—C4—C5	0.7 (6)	C31—N13—C27—C28	1.7 (5)
N1—C7—C8—N2	−4.0 (5)	C32—N14—C33—N15	0.1 (4)
N1—C7—C8—N4	176.7 (3)	C32—N14—C33—C34	178.9 (4)
N3—N4—C8—N2	0.6 (4)	C33—N14—C32—N16	0.3 (4)
N3—N4—C8—C7	179.9 (3)	C33—N14—C32—C31	−178.4 (3)
N4—N3—C9—N2	−0.1 (4)	C33—N15—N16—C32	0.6 (4)
N4—N3—C9—C10	−179.7 (4)	S1—C35—C36—C37	178.8 (3)
N5—C11—C12—C13	−0.9 (6)	S1—C35—C40—C39	−179.3 (4)
N5—C15—C16—N6	−6.1 (5)	O5—S1—C35—C36	36.1 (4)
N5—C15—C16—N8	172.2 (4)	O5—S1—C35—C40	−146.0 (3)
N7—N8—C16—N6	0.1 (5)	O6—S1—C35—C36	154.5 (3)
N7—N8—C16—C15	−178.2 (4)	O6—S1—C35—C40	−27.6 (4)
N8—N7—C17—N6	0.0 (6)	O7—S1—C35—C36	−86.8 (4)
N8—N7—C17—C18	−179.9 (4)	O7—S1—C35—C40	91.1 (4)
N9—C19—C20—C21	1.0 (7)	C35—C36—C37—C38	0.4 (6)
N9—C23—C24—N10	3.5 (5)	C36—C35—C40—C39	−1.3 (7)
N9—C23—C24—N12	−174.8 (4)	C36—C37—C38—C39	−1.3 (6)
N11—N12—C24—N10	0.1 (4)	C36—C37—C38—C41	177.9 (4)
N11—N12—C24—C23	178.6 (3)	C37—C38—C39—C40	0.8 (7)
N12—N11—C25—N10	0.1 (4)	C38—C39—C40—C35	0.5 (8)
N12—N11—C25—C26	−179.1 (3)	C40—C35—C36—C37	0.9 (6)
N13—C27—C28—C29	−1.1 (6)	C41—C38—C39—C40	−178.4 (4)
N13—C31—C32—N14	1.8 (5)	S2—C42—C43—C44	179.2 (3)
N13—C31—C32—N16	−176.8 (3)	S2—C42—C47—C46	−179.0 (3)
N15—N16—C32—N14	−0.5 (4)	O8—S2—C42—C43	−114.5 (4)
N15—N16—C32—C31	178.1 (3)	O8—S2—C42—C47	63.3 (4)
N16—N15—C33—N14	−0.5 (4)	O9—S2—C42—C43	8.7 (4)
N16—N15—C33—C34	−179.3 (4)	O9—S2—C42—C47	−173.5 (3)
C3—N1—C7—C6	0.3 (5)	O10—S2—C42—C43	126.1 (3)
C3—N1—C7—C8	−179.6 (3)	O10—S2—C42—C47	−56.2 (3)
C3—C4—C5—C6	0.0 (6)	C42—C43—C44—C45	−0.3 (7)
C4—C5—C6—C7	−0.6 (6)	C43—C42—C47—C46	−1.2 (6)
C5—C6—C7—N1	0.4 (6)	C43—C44—C45—C46	−1.0 (7)
C5—C6—C7—C8	−179.7 (3)	C43—C44—C45—C48	179.2 (4)
C6—C7—C8—N2	176.1 (3)	C44—C45—C46—C47	1.2 (6)
C6—C7—C8—N4	−3.2 (6)	C45—C46—C47—C42	−0.1 (6)
C7—N1—C3—C4	−0.9 (5)	C47—C42—C43—C44	1.4 (6)
C8—N2—C9—N3	0.4 (4)	C48—C45—C46—C47	−179.0 (4)

### Hydrogen-bond geometry (Å, º)

**Table:**

*D*—H···*A*	*D*—H	H···*A*	*D*···*A*	*D*—H···*A*
N3—H3···O10^i^	0.86	1.95	2.766 (4)	159
N7—H7···O6^ii^	0.86	2.34	3.064 (5)	142
N7—H7···O7^ii^	0.86	2.34	3.141 (6)	154
N11—H11···O9^iii^	0.86	1.92	2.769 (4)	170
N15—H15···O5^iv^	0.86	1.99	2.825 (4)	163
C4—H4···O2*W*^v^	0.93	2.48	3.383 (5)	165
C11—H11*A*···O5*W*	0.93	2.49	3.206 (8)	134
C28—H28···O4*W*^vi^	0.93	2.54	3.421 (6)	159
O2*W*—H2*WA*···O4	0.85	2.10	2.949 (4)	174
O2*W*—H2*WB*···O5	0.86	1.99	2.838 (4)	172
O4*W*—H4*WA*···O1	0.87	2.34	3.123 (5)	150
O4*W*—H4*WA*···O3	0.87	2.25	3.037 (4)	151
O4*W*—H4*WB*···O10	0.87	1.92	2.788 (5)	174
O5*W*—H5*WA*···O4*W*^vi^	0.86	1.98	2.810 (11)	159
O5*W*—H5*WB*···O4*W*	0.86	2.28	2.850 (10)	123
C13—H13···O8^vi^	0.93	2.57	3.256 (5)	131
C21—H21···O7^v^	0.93	2.44	3.280 (6)	150

Symmetry codes: (i) −*x*, −*y*+1, −*z*+1; (ii) −*x*, −*y*, −*z*; (iii) −*x*+1, −*y*+2, −*z*+1; (iv) −*x*+1, −*y*+1, −*z*; (v) −*x*, −*y*+1, −*z*; (vi) −*x*+1, −*y*+1, −*z*+1.
